# A phase II randomized trial of Observation versus stereotactic ablative RadiatIon for OLigometastatic prostate CancEr (ORIOLE)

**DOI:** 10.1186/s12885-017-3455-6

**Published:** 2017-06-29

**Authors:** Noura Radwan, Ryan Phillips, Ashley Ross, Steven P. Rowe, Michael A. Gorin, Emmanuel S. Antonarakis, Curtiland Deville, Stephen Greco, Samuel Denmeade, Channing Paller, Daniel Y. Song, Maximilian Diehn, Hao Wang, Michael Carducci, Kenneth J. Pienta, Martin G. Pomper, Theodore L. DeWeese, Adam Dicker, Mario Eisenberger, Phuoc T. Tran

**Affiliations:** 1 Department of Radiation Oncology & Molecular Radiation Sciences, The Sidney Kimmel Comprehensive Cancer Center, Johns Hopkins University School of Medicine, 1550 Orleans Street, CRB2 Rm 406, Baltimore, MD 21231 USA; 2 Department of Medical Oncology, The Sidney Kimmel Comprehensive Cancer Center, Johns Hopkins University School of Medicine, Baltimore, MD USA; 3 The James Buchanan Brady Urological Institute and Department of Urology, Johns Hopkins University School of Medicine, Baltimore, MD USA; 4 The Russell H. Morgan Department of Radiology and Radiological Science, Johns Hopkins University School of Medicine, Baltimore, MD USA; 5 Department of Radiation Oncology, Stanford University, Stanford, CA USA; 6 Department of Radiation Oncology, Thomas Jefferson University, Philadelphia, PA USA

**Keywords:** Prostate cancer, Stereotactic body radiation therapy, Stereotactic ablative radiotherapy, Oligometastasis

## Abstract

**Background:**

We describe a randomized, non-blinded Phase II interventional study to assess the safety and efficacy of stereotactic ablative radiotherapy (SABR) for hormone-sensitive oligometastatic prostate adenocarcinoma, and to describe the biology of the oligometastatic state using immunologic, cellular, molecular, and functional imaging correlates. 54 men with oligometastatic prostate adenocarcinoma will be accrued. The primary clinical endpoint will be progression at 6 months from randomization with the hypothesis that SABR to all metastases will forestall progression by disrupting the metastatic process. Secondary clinical endpoints will include local control at 6 months post-SABR, toxicity and quality of life, and androgen deprivation therapy (ADT)-free survival (ADT-FS). Further fundamental analysis of the oligometastatic state with be achieved through correlation with investigational ^18^F–DCFPyL PET/CT imaging and measurement of circulating tumor cells, circulating tumor DNA, and circulating T-cell receptor repertoires, facilitating an unprecedented opportunity to characterize, in isolation, the effects of SABR on the dynamics of and immunologic response to oligometastatic disease.

**Methods/design:**

Patients will be randomized 2:1 to SABR or observation with minimization to balance assignment by primary intervention, prior hormonal therapy, and PSA doubling time. Progression after 6 months will be compared using Fisher’s exact test. Hazard ratios and Kaplan-Meier estimates of progression free survival (PFS), ADT free survival (ADT-FS), time to locoregional progression (TTLP) and time to distant progression (TTDP) will be calculated based on an intention-to-treat. Local control will be assessed using Response Evaluation Criteria in Solid Tumors (RECIST) 1.1 criteria. Withdrawal from the study prior to 6 months will be counted as progression. Adverse events will be summarized by type and grade. Quality of life pre- and post- SABR will be measured by Brief Pain Inventory.

**Discussion:**

The ORIOLE trial is the first randomized, non-blinded Phase II interventional study in the North America evaluating the safety and efficacy of SABR in oligometastatic hormone-sensitive prostate cancer. Leading-edge laboratory and imaging correlates will provide unique insight into the effects of SABR on the oligometastatic state.

**Trial registrations:**

ClinicalTrials.gov Identifier: NCT02680587.

URL of Registry: https://clinicaltrials.gov/show/NCT02680587

Date of Registration: 02/08/2016.

Date of First Participant Enrollment: 05/23/2016.

## Background

Cancer is the second leading cause of death in the United States, chiefly from an inability to control metastatic disease. Systemic therapy alone is not curative for patients with most metastatic solid tumors [1]. The metastatic capacity of cancers behaves along a spectrum of disease progression, such that some tumors have spread widely before clinical detectability and others rarely if ever metastasize. The presence of an oligometastatic state, at which point metastases are limited in number and location, was originally proposed by Hellman and Weichselbaum, who suggested that these patients would benefit from effective local therapy in addition to systemic therapy [1].

The treatment of metastases depends on multiple factors including 1) the location of the primary tumor, 2) the size, number and location of metastases, 3) the availability and effectiveness of therapies (e.g. surgery, radiation, and chemotherapy), and 4) the patient’s functional status. Conventional moderate dose radiation for metastatic disease is given primarily for palliation, but recent advances in radiation delivery now make it possible to image and treat precisely within any anatomic region of the body [2, 3]. As a result, highly accurate radiation at tumorocidal doses can be delivered in 1 to 5 outpatient treatments [4–8].

Stereotactic radiation therapy entails highly conformal and precisely targeted radiation delivered in a very dose intensive fashion. In the brain, this approach (termed stereotactic radiosurgery or SRS) has been shown to be a highly effective treatment for brain metastases [9]. Data suggests that select small extracranial tumors (either primary or metastatic tumors) may be effectively controlled using a similar approach known as stereotactic body radiotherapy (SBRT) or stereotactic ablative radiotherapy (SABR). Local control in excess of 75% has been reported for metastatic tumors of the spine, lung and liver, which is significantly higher than standard conventional moderate dose radiation [5, 7, 8, 10–23]. Toxicity has been minimal in multiple U.S., European, and Japanese trials of SABR to the lung, liver, spine, pelvis and abdomen despite the use of very high biological equivalent doses for patients with both organ-confined and metastatic cancer.

The natural history of hormone sensitive oligometastatic prostate cancer is under studied. However, much is known regarding the preceding state of biochemically recurrent prostate cancer that has failed primary treatment. The management of this heterogeneous group of men with a rising PSA often involves relatively long periods of observation until metastases develop at which time the initiation of androgen deprivation therapy (ADT) is typically recommended. Although not entirely appropriate for all men with biochemical failure, data would suggest stalling initiation of ADT is not likely overtly detrimental to overall survival [24]. In the modern era with conventional imaging, oligometastatic hormone sensitive prostate cancer likely comprises a large number of men, possibly the majority of men following failed primary therapy [25–28]. Assuming these men are at a potentially curable state before castration-resistance develops, we need additional treatment strategies to re-examine this large cohort of men.

Based on this emerging evidence, we propose a phase II study of SABR in patients with oligometastatic hormone sensitive prostate cancer. This study is designed to determine if we can improve the outcome of prostate cancer in these men and also to advance the basic understanding of the oligometastatic state as it pertains to signaling dynamics, cell biology, and immunologic responses. Clinically, we anticipate that SABR in the oligometastatic setting will safely forestall disease progression, thereby lengthening the time before initiation of hormonal therapy and protecting patients from the known deleterious side effects of this conventional systemic approach and thus improve quality of life [24].

As we continue to refine the standard approaches to treatment of oligometastatic cancer within and beyond the prostate, principle questions remain unanswered which may greatly enhance our collective ability to improve patient outcomes. Chief among these are how best to identify patients in the oligometastatic state, and what aspects of this state differentiate it fundamentally from patients with organ-confined or polymetastatic disease. To address the former, PET/CT imaging utilizing the investigational prostate specific membrane antigen (PMSA) targeted radiotracer, ^18^F-DCFPyL, will be compared to conventional bone scan and CT imaging to assess the utility of this imaging test in identifying oligometastases before SABR and monitoring disease response following SABR [29–31]. Alterations in the biology of the oligometastatic state induced by SABR will be investigated using leading-edge correlatives, including: analysis of circulating tumor cells (CTCs; Epic Sciences, San Diego, CA), deep sequencing of circulating tumor DNA (ctDNA) using Cancer Personalized Profiling by deep sequencing (CAPP-Seq) to non-invasively assess tumor burden, and ImmunoSEQ profiling of T-cell repertoires to elucidate the immunological response to SABR (Adaptive Technologies, Seattle, WA). Finally, use of the Color Genomics platform (Burlingame, CA), a hereditary cancer assay assessing pathogenic mutations in 30 cancer predisposition genes that account for >90% of the germline mutations known to occur in men with castrate resistant metastatic prostate cancer (mCRPC), will inform efforts to advance a more personalized medicine approach to tailor screening and therapies to these men [32, 33].

## Methods/design

This study was approved by the Reaserach Ethics Boards of Johns Hopkins Medicine. The ORIOLE Trial is registered at the US National Institutes of Health (ClinicalTrials.gov) # NCT02680587 and Current Controlled Trials IND/IDE Number: 121064.

### Objectives

Primary endpoint: to determine the proportion of men with oligometastatic hormone sensitive prostate cancer who have progressed after 6 months from randomization to observation *versus* SABR.

#### Secondary endpoints

- To describe the toxicity of SBRT/SABR delivered for the population enrolled using Common Terminology Criteria for Adverse Events (CTCAE) version 4.0.
- To determine local control at 6-months after SABR in patients with oligometastatic disease.
- To assess progression free survival (PFS) and ADT-free survival (ADT-FS) after randomization defined as the time interval between the day of randomization and progression.
- To assess quality of life in the SBRT/SABR arm using the Brief Pain Inventory form [34].
- To estimate the proportion of ^18^F-DCFPyL-PET/MRI or –PET/CT positive sites that are positive for new or progressive metastatic disease by bone scan/CT at baseline and 6 months following SABR and vice versa.
- To enumerate CTCs using EPIC HD-CTC platforms at baseline and day 180 from randomization.
- To enumerate circulating tumor DNA (ctDNA) using Cancer Personalized Profiling by deep sequencing (CAPP-Seq) at baseline, day 90 and day 180 from randomization for control and SABR arms.
- To quantitatively sequence T-cell receptor (TCR) repertoires using peripheral blood monocytes and the ImmunoSEQ platform at baseline and day 90 from randomization.

#### Inclusion criteria

- Patient must have 1-3 asypmtomatic metastatic tumor(s) of the bone or soft tissue developed within the past 6-months that are ≤ 5.0 cm or < 250 cm^3.^
- Patient must have had their primary tumor treated with surgery and/or radiation and salvage radiation to the prostate bed or pelvis is allowed.
- Histologic confirmation of malignancy (primary or metastatic tumor).
- Prostate specific antigen (PSA) ≥ 0.5 ng/mL but ≤ 50 ng/mL and Testosterone ≥ 125 ng/dL.
- PSA doubling time (PSADT) < 15 months. PSADT will be calculated using as many PSA values that are available from time of relapse (PSA > 0.2 ng/dL).
- Patient may have had prior systemic therapy and/or ADT associated with treatment of their primary prostate cancer. Patient may have had ADT associated with salvage radiation therapy.
- Patient must be ≥ 18 years of age, have the ability to understand, and the willingness to sign, a written informed consent document.
- Patient must have an Eastern Cooperative Oncology Group performance status ≤ 2.
- Patient must have normal organ and marrow function as defined as:
  - Leukocytes >2,000/μL, absolute neutrophil count >1,000/μL, platelets >50,000/μL

#### Exclusion criteria

- No more than 3 years of ADT is allowed, with the most recent ADT treatment having occurred greater than 6 months prior to enrollment.
- ^18^F-DCFPyL-PET/MRI or ^18^F-DCFPyL-PET/CT scan within the past 6 months with results that demonstrate lesions not seen on baseline CT or bone scan
- Castration-resistant prostate cancer (CRPC).
- Spinal cord compression or impending spinal cord compression.
- Suspected pulmonary and/or liver metastases (greater > 10 mm in largest axis).
- Receipt of any other investigational agents or participation in a concurrent treatment protocol.
- Serum creatinine and total bilirubin > 3 times the upper limit of normal
- Liver Transaminases > 5-times the upper limit of normal.
- Inability to lie flat during or tolerate PET/CT, PET/MRI or SABR.
- Refusal to sign informed consent.

### Evaluation of randomization and blinding

This study will employ a randomized phase II design to determine the appropriateness of a subsequent phase III trial based on comparison of rate of progression at 6 months. An interactive web response system (IWRS) will be utilized to obtain the patient’s randomization assignment. Randomization will occur in a 2:1 fashion for SABR and observation arms, respectively. A minimization approach [35] will be applied to ensure balanced assignment to each treatment arm by: 1) Initial treatment with surgery vs. radiation therapy; 2) Prior hormonal therapy vs. no prior hormonal therapy; and 3) PSADT <6 mos vs. 6-14.9 mos. The on-study date for protocol entry will be the day that the study subject is randomized. Patients will be re-evaluated for radiographic response 6 months after randomization. Trial radiologists evaluating for treatment responses will be blinded to the treatment group and treatment specifics. The trial radiologists and the principal investigator will be unblinded only if a patient progresses at any time during the study.

### Interventions

**Fig. 1:**
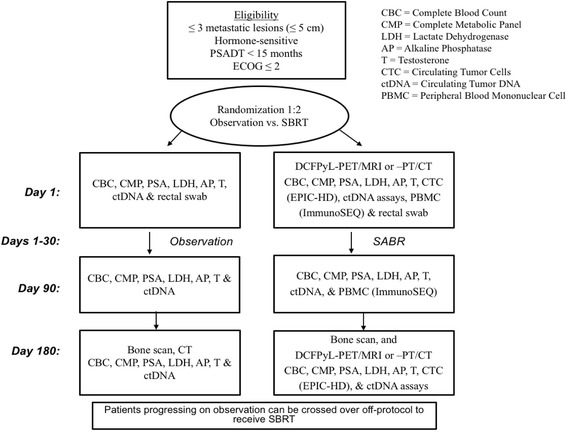
ORIOLE Study Schema. Subjects who meet eligibility criteria and qualify for enrollment will be stratified and randomized as demonstrated

Eligibility work-up will include a complete blood count, serum chemistries, PSA, and radiographic studies (of involved sites) and bone scan. Subjects who meet eligibility criteria and qualify for enrollment will be stratified and randomized (Fig. 1).

The following is a detailed outline, after randomization, involving each study arm:

1. Observation Arm: Active Clinical Surveillance

- Defined with 3 time points, involving the following:

Day 1

- Standard blood tests [complete blood count with differential (CBC w/Diff), lactate dehydrogenase (LDH), serum chemistry, and PSA evaluation]
- Research laboratory tests: CAPP-Seq, rectal swab, and Color Test

Day 90

- Physician examination, medical history, medication review, performance status, quality of life (QoL) and adverse event (AE) evaluations
- Standard blood tests (CBC w/Diff, LDH, serum chemistry, testosterone, and PSA evaluation)
- Research blood tests: CAPP-Seq

Day 180

- Physician examination, medical history, medication review, performance status, QoL and AE evaluations
- Standard blood tests (CBC w/Diff, LDH, serum chemistry, testosterone and PSA evaluation),
- Research blood tests: CAPP-Seq
- Imaging: bone scan and CT/MRI

2. SABR Arm:

- Defined with 3 time points, involving the following:
  Day 1
  Standard blood tests (CBC w/Diff, LDH, serum chemistry, and PSA evaluation)
    Research laboratory tests: EPIC HD-CTC, ImmunoSEQ, CAPP-Seq, rectal swab, and Color Test
  Imaging: ^18^F-DCFPyL PET/CT or PET/MRI
  Day 90
  Physician examination, medical history, medication review, performance status, QoL and AE evaluations
  Standard blood tests (CBC w/Diff, LDH, serum chemistry, testosterone, and PSA evaluation)
  Research blood tests: ImmunoSEQ, CAPP-Seq
  Day 180
  Physician examination, medical history, medication review, performance status, QoL and AE evaluations
  Standard blood tests (CBC w/Diff, LDH, serum chemistry, testosterone, and PSA evaluation)
  Research blood tests: EPIC HD-CTC, CAPP-Seq
  Imaging: Bone scan and ^8^F-DCFPyL PET/CT or PET/MRI
- Research Imaging
^18^F-DCFPyL PET/CT or PET/MRI images will be evaluated and compared to bone scan. However, additional sites(s) of suspected metastatic disease detected using ^18^F-DCFPyL will not be considered for treatment by SABR or undergo further evaluation. The results of PSMA-targeted PET imaging will not be made available to the treating physicians until after the trial is completed and these results will not be made available to the patient.
- SBRT/SABR Planning and Dosage:

- CT- and/or MRI-simulation will be performed with fabrication of a radiation therapy immobilization device custom-made for each patient. The treating radiation oncologist will identify the location of the tumor. Gross tumor volume (GTV) delineation will be performed with a diagnostic radiologist on sequential axial computed tomography images. A radiosurgical treatment plan will then be developed based on tumor geometry and location. The clinical tumor volume (CTV) will equal the GTV. The dose will be prescribed to the minimal isodose line that completely covers the planning target volume (PTV), defined as CTV plus a variable (up to 5 mm) margin. Adjacent normal structures, including but not limited to the heart, esophagus, aorta, spinal cord, kidneys, rectum, bowel, liver, and stomach, within 5 cm of the CTV will be identified for the purpose of limiting incidental radiation to these structures.
- In addition, prior to treatment delivery, a four-dimensional cone beam CT study will be performed on individual patients to assess respiration and determine targeting accuracy for tumors that may be subject to respiratory motion such as those in the bones of the thorax. If tumor motion is greater than 5 mm, the PTV will be expanded accordingly.
- SABR will be delivered in 1 to 5 fractions, and the dose and fractionation schedule will depend on the size and location of the lesion and the surrounding normal tissue constraints in accordance with AAPM Task Group 101 recommendations [36].
- Within three weeks of the initial treatment planning imaging study, SABR will be administered using image-guidance. During treatment, real time cone beam CT images of the patient’s body site of interest will be obtained. Cone beam CT will be obtained immediately prior to treatment and will be repeated until the treatment shift, required to align the planning CT and the cone beam CT performed on the day of treatment, is within tolerance for the body site.

d. Early Stopping Guidelines

- Site-specific grade 4/5 toxicity will be monitored in the SABR arm. If it becomes evident that the proportion of grade 4/5 toxicity at specific sites convincingly exceeds 20%, the study will be halted for a safety consultation. Specifically, we will apply a Bayesian toxicity monitoring rule that suspends the enrollment for a posterior probability ≥ 75% of toxicity being larger than 20%. The monitoring rule uses Beta (0.5, 5.5) as prior distribution, meaning our prior guess of the proportion of toxicity is 8.3% with a 90% chance that this proportion is 0.04%-30.6%.

Follow-Up:

- Patients will be followed from Day 1 to Day 180.
- All AEs and serious adverse events (SAEs) are recorded on source documents. The investigator will follow up on all AEs and SAEs until the events have subsided, returned to baseline or, in case of permanent impairment, until the condition stabilizes.

### Stastical analysis

#### Sample size and accrual

The primary endpoint will be progression of disease at 6 months. Historical data on this patient population indicate that >80% would show progression, within a 6-month period without treatment, and thus this is the progression rate we expect in subjects in the control/observation arm [37–39]. We hypothesize that SABR will reduce progression at 6 months by 50% [40]. A sample size using a 2:1 randomization scheme of 36 in the SABR arm and 18 patients in the control group will provide 85% power to detect a decrease in relapse rate from 80% to 40% with a type I error = 0.05 using one-sided Fisher’s exact test. Thus, we will accrue a total of 54 patients. Patients withdrawing within one month of enrollment or prior to day 1 of SABR or observation will be replaced.

#### Data analysis

##### 2.4.2.1.Analysis of primary objective

- A minimization approach [35] will be applied to ensure balanced assignment to each treatment arm by: 1) Initial treatment with surgery vs. radiation therapy; 2) Prior hormonal therapy vs. no prior hormonal therapy; and 3) PSADT <6 mos vs. 6-14.9 mos. Baseline PSA level is defined as that measured Day 1 following randomization.
- The primary clinical outcome will be the proportion of patients who have progressed after 6 months from randomization. For each arm, we will calculate the proportion of patients who have progressed and extract 95% confidence intervals. If a patient has withdrawn from the study before 6 months, they will be considered to have progressed.
- Progression will be a composite endpoint defined from the Prostate Cancer Working Group 2 (PCWG2) criteria for mCRPC [38] and our previous trials in a population of men with biochemical failure without metastases [37–39]. Progression will be defined as either: 1) a ≥ 25% increase in PSA from nadir (and by ≥ 2 ng/mL), requiring confirmation ≥ 4 weeks later (PCWG2 criteria); and/or, 2) clinical/radiographic-progression defined as symptomatic progression (worsening disease-related symptoms or new cancer-related complications), or radiologic progression (on CT scan: ≥ 20% enlargement in sum diameter of soft-tissue target lesions [RECIST 1.1 criteria]; on bone scan: ≥ 1 new bone lesions), initiation of ADT or death due to any cause, whichever occurs first. Death is considered a severe adverse event here.
- We will compare the proportion of patients who have progressed in the observation and SABR arms using Fisher’s exact test. The analysis population includes all randomized subjects based on intention-to-treat.

##### 2.4.2.2.Analysis of secondary objectives

- For safety analysis, adverse events will be summarized by type and grade.
- Hazard rate estimates and 95% confidence intervals as well as Kaplan-Meier (KM) estimates will be calculated for PFS, ADT-FS, TTLP and TTDP. The median PFS, ADT-FS, TTLP and TTDP will be reported.
- Each metastatic lesion will be considered a target lesion and independently evaluated for response using RECIST 1.1 or bone scan evaluation criteria below. The lesion will be coded as locally controlled if it is considered stable radiographic disease or if there is evidence of a partial or complete response. Local control assessment will start at three months following randomization and continuous assessment will be pursued during the follow-up period. The proportion of locally controlled lesions will be estimated using generalized estimating equations.
- QoL will be assessed using the Brief Pain Inventory form. An overall score will be calculated pre-treatment and at the time of the 2^nd^ radiologic reassessment. The change in score will be evaluated by paired t-test.

#### Response criteria

- Evaluation of Target Lesions and PSA Response

- Complete Response (CR): Disappearance of all target lesions and PSA below baseline
- Partial Response (PR): At least a 30% decrease in the sum of the longest diameter (LD) of target lesions, taking as reference the baseline sum OR at least 1/3 of lesions are stable or resolved by bone scan AND PSA below baseline
- Progressive Disease (PD): At least a 20% increase in the sum of the LD of target lesions, taking as reference the smallest sum LD recorded since treatment initiation OR the appearance of >1 new lesion(s) by bone scan OR PSA ≥25% above nadir or > 50 ng/ml.
- Stable Disease (SD): Neither sufficient shrinkage to qualify for PR nor sufficient increase to qualify for PD, taking as reference the smallest sum LD since treatment initiation OR PSA > baseline but not ≥25% above nadir and <50 ng/ml.
- Complete Response (CR): Disappearance of all target lesions and PSA below baseline
- Partial Response (PR): At least a 30% decrease in the sum of the longest diameter (LD) of target lesions, taking as reference the baseline sum OR at least 1/3 of lesions are stable or resolved by bone scan AND PSA below baseline
- Progressive Disease (PD): At least a 20% increase in the sum of the LD of target lesions, taking as reference the smallest sum LD recorded since treatment initiation OR the appearance of >1 new lesion(s) by bone scan OR PSA ≥25% above nadir or > 50 ng/ml.
- Stable Disease (SD): Neither sufficient shrinkage to qualify for PR nor sufficient increase to qualify for PD, taking as reference the smallest sum LD since treatment initiation OR PSA > baseline but not ≥25% above nadir and <50 ng/ml.

b. Evaluation of Best Overall Response: The best overall response is the best response recorded between treatment initiation and disease progression/recurrence
c. Duration of Response: Response will be defined as evidence of CR, PR, or stable disease.

- Duration of CR or PR: The duration of CR or PR will be recorded from the date criteria for CR or PR are first met until the first date current or progressive disease is objectively documented (taking as reference for progressive disease the smallest measurements recorded since treatment initiation).
- Duration of Stable Disease: Stable disease will be recorded until the criteria for progression are met, taking as reference the smallest measurements recorded since the treatment started.

## Discussion

The standard treatment options for metastatic hormone sensitive prostate cancer have remained unchanged for many years involving principally hormonal therapy. However, hormonal therapy can have troublesome side effects and any effort to delay the start of hormonal therapy would be an advantage to the patient. Radiation treatment was historically not given at high enough doses to metastases to provide durable local control. SABR is highly targeted radiation, delivered in a dose-intensive fashion in 1 to 5 fractions, which has been shown to be very effective on bone and soft tissue metastases. This phase II randomized study will compare SABR to observation with respect to progression of disease, freedom from hormonal therapy, and other relevant clinical endpoints. Simultaneously, a unique perspective on the impact of SABR on the biology of the oligometastatic state will be obtained through correlation of clinical response with measures of tumor burden, hematologic dynamics of metastasis, and immunologic response. Finally, the pursuit of patient-centered, personalized approaches to treatment will be furthered through investigation of targeted imaging and genomic susceptibility characterization.

## Abbreviations

ADL: Activities of daily living
ADT: Androgen deprivation therapy
AE: Adverse event
BID: Twice daily
BSA: Body surface area
CBC: Complete blood count
CI: Confidence interval
CMAX: Maximum concentration of drug
CNS: Central nervous system
CR: Complete response
CRF: Case report/Record form
CRPC: Castration-Resistant Prostate Cancer
CTC: Circulating Tumor Cell
CTCAE: Common Terminology Criteria for Adverse Events
DLT: Dose Limiting Toxicity
DSMB: Data Safety Monitoring Board
ECG: Electrocardiogram
GI: Gastrointestinal
Hgb: Hemoglobin
HIV: Human Immunodeficiency Virus
HPF: High-power field
HTN: Hypertensions
IRB: Institutional Review Board
IV: Intravenous
LLN: Lower limit of normal
MTD: Maximum tolerated dose
OS: Overall survival
PD: Progressive diseased
PFS: Progression free survival
PLT: Platelet
PR: Partial response
PSADT: PSA Doubling Time
QD: Once daily
RECIST: Response evaluation criteria in solid tumors
RR: Response rate
SABR: Stereotactic ablative radiation therapy
SAE: Serious adverse event
SBRT: Stereotactic body radiation therapy
SD: Stable disease
TTP: Time to progression
ULN: Upper limit of normal
UNK: Unknown
WBC: White blood cell
